# The Application and Advantages of Celluloid-Ring Pessaries, as Made by the Rhenish Rubber and Celluloid Factory, Mannheim

**Published:** 1890-01

**Authors:** Bernhard Sigmund Schultze

**Affiliations:** 2 Sellierstrasse; Jena, Germany


### THE APPLICATION AND ADVANTAGES OF CELLULOID-RING PESSARIES, AS MADE BY THE RHENISH RUBBER AND CELLULOID FACTORY, MANNHEIM.

BY Dr. BERNHARD SIGMUND SCHULTZE, Jena, Germany.

IT IS a fact that at the present time much dexterity is required in the selection of a suitable pessary out of a number ready-made to shape. The position of the uterus has been commented on by Marion Sims, in his "Clinical Notes on Uterine Surgery," London, 1866. He compares the process with that of a dentist striving to fit every patient with the same plate.

The individual differences in every vagina, and the mechanical varieties of pathological conditions which call for the use of a pessary, are in fact so numerous, that it is but just that every case should have its own special pessary.

Marion Sims made his pessaries out of tin, to which was added some lead. Following the example of Marion Sims, I caused some soft copper-wire pessaries with soft-rubber coating to be made—which have found wide application. The tin, like the copper-wire pessaries, may be moulded into any form to suit the conditions of each individual case. A certain skill is necessary, however, to ascertain the wants of each case, and to shape the pessary accordingly.

Better than the tin and wire-rubber rings for vaginal pessaries, are the celluloid rings,—made after my designs by the Rhenish rubber and celluloid factory, in Mannheim (Germany). Placed in hot water, they become pliable, and when once moulded and cooled, retain their shape; they are much lighter than either the tin or wire rings, are more smooth than the wire pessaries and remain so, are not acted on by the secretions of the vagina or uterus. Celluloid pessaries do not irritate the vaginal mucous membrane, do not cause any secretion and no bad odor, provided that the uterus has been placed in its normal position beforehand, that the indications for the use of a pessary are present, that the correct size of the rings has been chosen, and that the ring has been moulded to adapt itself to the special case.

Celluloid rings combine the advantages of tin and wire-rubber pessaries in their *adaptibility to every individual case*, with those of hard rubber pessaries in the *durability and perpetual smoothness of their surface.*

To mould the celluloid rings, they must be placed in boiling water; after two or three minutes they are sufficiently pliable to assume any form, and are then placed in cold water where they become firm and rigid.

Retroflexion, retroversion, and prolapse of the uterus, are the displacements amenable to treatment with these pessaries.

It is not simply the introduction of a pessary which corrects the position of the uterus; no pessary is capable of doing this. In all cases it is necessary to first place the uterus in its normal position; if retroflected, it should be brought anteriorly. This is done most satisfactorily by manipulation with one hand in the vagina and the other on the abdomen, care being taken to have the bladder empty. The pessary may then be introduced with a good chance of success. It fulfills the office of retaining the uterus in situ, without interfering with any of its normal movements.

The pessaries which have proven most successful in correcting retroflexion and prolapse are the figure 8 form, the sled form, the Hodge, and Gaillard Thomas's modification of the Hodge form.

In the great majority of cases of retroflexion, the figure 8 form has proven most successful. With simultaneous prolapse of the anterior vaginal wall, the sled form is often more successful. In cases of long and rigid vaginas, the Hodge form is preferable; in very long and flaccid vaginas, with relaxation of the posterior wall, the Thomas pessary renders the best service. The figure 8 form and Hodge pessary, with many modifications are the ones most employed by me.

The celluloid rings may be moulded to any form of vaginal pessary the physician may prefer.

The pessary must not protrude outside the vagina; the consequent entrance of air causes vaginitis and endometritis, with their well-known disturbances.

Normal, or nearly normal, firmness and elasticity of the perineum are requisites for the successful operation of a pessary. Where the perineum has been lacerated, or become flaccid, an operation may, perhaps, be necessary to restore it to its former condition.

For more detailed descriptions on the use of pessaries, and displacements of the uterus, see my paper on "Zur Klarstellung der Indicationen für Behandlung der Ante—und Retroversionen und Flexionen der Gebärmutter. Sammlung Klinischer Vorträge von Volkmann No. 176. Leipzig, Breitkopf u. Härtel, 1879," and in "Pathologie und Therapie der Lageveränderungen der Gebärmutter, Berlin, Hirschwald, 1881;" also, "Traitê des Dêviations Utêrines," Paris, Octave Doin, 1884; and in "The Pathology and Treatment of Displacements of the Uterus," London, H. K. Lewis, 1888.

2 SELLIERSTRASSE.

